# Supplementary material for: Turmeric and Cumin Instead of Stock Cubes: An Internet Survey of Spices and Culinary Herbs Used in Poland Compared with Historical Cookbooks and Herbals
Source: Plants (Basel). 2023 Jan 29;12(3):591. doi: 10.3390/plants12030591 (PMC9919365; doi:10.3390/plants12030591)
Supplement: Supplementary file 1 [file plants-12-00591-s001.zip › plants-2164561-supplementary.pdf]

Supplementary Materials

1 Płeć

a. Kobieta
b. Mężczyzna
c. Inna
d. Nie chcę odpowiadać na to pytanie

2 Wiek w latach

……

3 Wykształcenie

a. Podstawowe
b. Gimnazjalne
c. Średnie
d. Zawodowe
e. Wyższe

Inne…….

4 Miejsce zamieszkania

a. Miasto powyżej 100 tysięcy mieszkańców
b. Miasto 100-51 tysięcy mieszkańców
c. Miasto 21 – 50 tysięcy mieszkańców
d. Wieś lub miasto do 20 tysięcy mieszkańców

5 Województwo, w którym mieszkasz

……

6 Dochód miesięczny

a. Jestem osobą majętną
b. Jestem osobą przeciętną jeśli chodzi o dochody
c. Jestem osobą o małych dochodach

7 Jak często sam przygotowujesz posiłki w domu

a. Codziennie
b. Przynajmniej raz w tygodniu
c. Rzadziej niż raz w tygodniu
d. W ogóle nie gotuję

8 Czy pojawiają się w twoim jadłospisie nowe, dotąd nieobecne potrawy?

a. Tak, często
b. Tak, ale dość rzadko
c. Bardzo rzadko
d. Nigdy

9 Czy stosujesz w kuchni przyprawy, poza solą i pieprzem czarnym

a. Tak, dużo
b. Tak, ale niewiele
c. Czasami, jeśli są wymagane w przepisie
d. Nie

10 Jeśli w powyższym pytaniu wybrałeś „nie", napisz dlaczego.

………

11 Czym kierujesz się przy kupnie przypraw (możesz wybierać więcej niż jedną odpowiedź)

a. Walorami smakowymi
b. Aromatem
c. Ciekawością
d. Modą
e. Zaleceniami zdrowotnymi
f. Rekomendacjami innych
g. Nie zastanawiałam się nas tym – zawsze kupuję te same lub nie kupuję ich wcale

Inna odpowiedź………

12 Czy uważasz, ze przyprawy są niezbędne w nadawaniu potrawie smaku

a. Tak
b. Nie
c. To zależy od potrawy
d. Nie wiem

13 Czy uważasz, że przyprawy mają właściwości lecznicze?

a. Tak
b. Tylko niektóre
c. Nie wiem
d. Nie

14 Czy w swojej diecie stosujesz przyprawy w celu poprawy zdrowia?

a. Tak – widzę pozytywny efekt
b. Tak – nie widzę pozytywnego efektu
c. Nie jestem chory, ale przyprawy stosuję profilaktycznie
d. Nie – uważam, że przyprawy mnie nie uleczą

Inna odpowiedź………

15 Wymień trzy przyprawy, które według ciebie mają właściwości lecznicze (o ile takie znasz). Podaj ich zdrowotne działanie. Jeśli nic o tym nie wiesz, pomiń to pytanie.

………

16 Wymień przyprawy, których uważasz przynajmniej raz w tygodniu.

………

17 Jakich przypraw używasz rzadziej niż raz w tygodniu, ale przynajmniej raz w roku?

……

18 Których z przypraw używasz dopiero od ostatnich kilku lat (10 lat temu nie było ich w twojej kuchni)?

………

19 Czy są przyprawy, których używałeś wcześniej niż 10 lat temu, a obecnie nie występują w twojej kuchni? Jeśli tak, to jakie?

……

20 Jak często podróżujesz za granicę?

a. Więcej niż 5 razy w roku
b. 4-2 razy w roku
c. 1 raz w roku
d. Nie bywam za granicą

21 Czy zacząłeś używać konkretnych przypraw po podróży do któregoś z krajów?

a. Tak
b. Nie
c. Nie pamiętam

22 Jeśli powyżej odpowiedziałeś „tak", napisz jakie odwiedziłeś kraje i jakich przypraw zacząłeś używać.

……

23 Jak często oglądasz programy kulinarne poświęcone kuchniom z innych kultur?

a. Systematycznie – minimum raz w tygodniu
b. Często – minimum raz w miesiącu
c. Sporadycznie
d. Nie oglądam

24 Jak często korzystasz z ofert restauracji etnicznych (oferujących dania z innych krajów)?

a. Systematycznie – minimum raz w tygodniu
b. Często – minimum raz w miesiącu
c. Sporadycznie
d. Nigdy

25 Jeśli korzystasz systematycznie lub często z restauracji etnicznych wymień z jakich.

……

26 Czy kiedyś mieszkałeś w innym kraju, gdzie nauczyłeś się używać nowych przypraw?

a. Tak
b. Nie

Jeśli tak, podaj kraj i przyprawy.

……

27 Czy stosujesz przyprawy w celu innych niż kulinarne lub lecznicze?

a. Tak
b. Nie
c. Nie wiem

28 Czy uprawiasz jakieś przyprawy w ogrodzie?

a. Tak
b. Nie, mimo że mam ogród
c. Nie, nie mam ogrodu

29 Jeśli uprawiasz przyprawy w ogrodzie, napisz jakie.

……

30 Czy uprawiasz jakieś przyprawy w domu (dłużej niż trzymanie przez kilka dni doniczki z ziołami z supermarketu)? Jeśli tak, to jakie?

……

31 Czasem roślinom zielnym i przyprawowym nadano magiczną moc. Czy uważasz, ze obecnie również są wykorzystywane w praktykach magicznych, czy w obrzędach religijnych?

a. Tak, stosuję sam/sama
b. Tak, ale nie robię tego sam/sama
c. Nie wiem
d. Nie

32 W jakich praktykach magicznych lub obrzędach religijnych stosujesz przyprawy? (jeśli to ciebie nie dotyczy, pomiń to pytanie).

……

33 Jaki jest twój stosunek do diety mięsnej?

a. Jem i zawsze jadłem mięso
b. Jem mięso, ale kiedyś przez dłuższy czas byłem na diecie wegetariańskiej
c. Jem ryby, ale nie jem innych mięs
d. Jestem wegetarianinem
e. Jestem weganinem
f. Jem mięso, ale chciałem przejść na wegetarianizm

34 Wymień źródła, z których czerpiesz informacje na temat zastosowania przypraw, możesz wymienić więcej niż jedno.

a. Od członków rodziny
b. Od przyjaciół i znajomych
c. Z książek kucharskich
d. Z podróży po świecie
e. Z informacji zamieszczanych na opakowaniu przypraw
f. Z internetu
